# SLO co-opts host cell glycosphingolipids to access cholesterol-rich lipid rafts for enhanced pore formation and cytotoxicity

**DOI:** 10.1128/mbio.03777-24

**Published:** 2025-01-21

**Authors:** Pooja Sanduja, Stefanie S. Schmieder, Buket Baddal, Songhai Tian, Jorge J. Velarde, Wayne I. Lencer, Min Dong, Michael R. Wessels

**Affiliations:** 1 Division of Infectious Diseases, Boston Children’s Hospital, Boston, Massachusetts, USA; 2 Department of Pediatrics, Harvard Medical School, Boston, Massachusetts, USA; 3 Division of Gastroenterology, Boston Children’s Hospital, Boston, Massachusetts, USA; 4 Harvard Digestive Diseases Center, Boston, Massachusetts, USA; 5 Department of Urology, Boston Children’s Hospital, Boston, Massachusetts, USA; 6 Department of Surgery, Harvard Medical School, Boston, Massachusetts, USA; 7 Department of Microbiology, Harvard Medical School, Boston, Massachusetts, USA; University of Oklahoma Health Sciences Center, Oklahoma City, Oklahoma, USA

**Keywords:** *Streptococcus pyogenes*, group A *Streptococcus*, streptolysin O, toxin, cholesterol-dependent cytolysin, lipid raft, glycosphingolipid, glycan, receptor

## Abstract

Streptolysin O (SLO) is a virulence determinant of group A *Streptococcus* (*S. pyogenes*), the agent of streptococcal sore throat and severe invasive infections. SLO is a member of a family of bacterial pore-forming toxins known as cholesterol-dependent cytolysins, which require cell membrane cholesterol for pore formation. While cholesterol is essential for cytolytic activity, accumulating data suggest that cell surface glycans may also participate in the binding of SLO and other cholesterol-dependent cytolysins to host cells. Here, we find that unbiased CRISPR screens for host susceptibility factors for SLO cytotoxicity identified genes encoding enzymes involved in the earliest steps of glycosphingolipid (GSL) biosynthesis. Targeted knockouts of these genes conferred relative resistance to SLO cytotoxicity in two independent human cell lines. Inactivation of *ugcg*, which codes for UDP-glucose ceramide glucosyltransferase, the enzyme catalyzing the first glycosylation step in GSL biosynthesis, reduced the clustering of SLO on the cell surface. This result suggests that binding to GSLs serves to cluster SLO molecules at lipid rafts where both GSLs and cholesterol are abundant. SLO clustering and susceptibility to SLO cytotoxicity were restored by reconstituting the GSL content of *ugcg* knockout cells with ganglioside GM1, but susceptibility to SLO cytotoxicity was not restored by a GM1 variant that lacks an oligosaccharide head group required for SLO binding, nor by a variant with a “kinked” acyl chain that prevents efficient packing of the ganglioside ceramide moiety with cholesterol. Thus, SLO appears to co-opt cell surface glycosphingolipids to gain access to lipid rafts for increased efficiency of pore formation and cytotoxicity.

**IMPORTANCE:**

Group A *Streptococcus* is a global public health concern as it causes streptococcal sore throat and less common but potentially life-threatening invasive infections. Invasive infections have been associated with bacterial strains that produce large amounts of a secreted toxin, streptolysin O (SLO), which belongs to a family of pore-forming toxins produced by a variety of bacterial species. This study reveals that SLO binds to a class of molecules known as glycosphingolipids on the surface of human cells and that this interaction promotes efficient binding of SLO to cholesterol in the cell membrane and enhances pore formation. Understanding how SLO damages human cells provides new insight into streptococcal infection and may inform new approaches to treatment and prevention.

## INTRODUCTION

Cholesterol-dependent cytolysins (CDCs) constitute a family of pore-forming toxins produced by many, predominantly Gram positive, species of bacteria (1). For several pathogens, CDCs are thought to contribute to virulence by damaging host tissues and inactivating immune cells (1, 2). The toxins may also stimulate host inflammatory responses by signaling through Toll-like receptors or by activating host inflammasomes (3–5). Examples of specialized functions identified for specific CDCs include the ability of listeriolysin O to mediate the escape of *Listeria monocytogenes* from the macrophage phagosome and the capacity of streptolysin O (SLO) to translocate its co-toxin NAD-glycohydrolase (NADase) into host cells during infection by group A *Streptococcus* (*S. pyogenes* or GAS) (6–8). Notwithstanding the multiple and varied functional roles of these toxins in bacterial interactions with their hosts, CDCs share structural features as well as the conserved function of pore formation on cholesterol-containing cell membranes. A general paradigm has emerged for CDC binding and pore formation based largely on studies of perfringolysin O (PFO), which binds to cholesterol in cell membranes, oligomerizes, and inserts as multimeric complexes to form large transmembrane pores (9).

The interaction of CDCs with membrane cholesterol is an essential step in pore formation for most if not all CDCs (10). However, there is accumulating evidence that for many CDCs, alternative or additional non-cholesterol receptors contribute to CDC binding to host cells. Giddings et al. (11) showed that depletion of cholesterol from human erythrocytes abrogated the binding of PFO but had minimal effect on the binding of intermedilysin (from *Streptococcus intermedius*) or SLO. Subsequent work by Giddings and colleagues identified the complement regulatory protein CD59 as a receptor on human cells for intermedilysin, but the non-cholesterol receptor(s) for SLO remained unknown (12). More recent studies by Shewell et al*.* found that eight major CDCs, including SLO, could bind oligosaccharides corresponding to glycans represented on various human cell surface glycoproteins, glycolipids, and polysaccharides (13, 14). Furthermore, some of the identified oligosaccharides were found to inhibit CDC-mediated hemolysis of human erythrocytes *in vitro*, providing evidence that suggests a biologically significant role for glycan receptors in CDC interaction with human cells. By glycan array and surface plasmon resonance analysis, SLO was observed to bind to a variety of oligosaccharides, many of which feature a terminal galactose residue.

GAS is a human-specific pathogen responsible for common localized infections such as pharyngitis and impetigo, a superficial skin infection, as well as less common but potentially life-threatening conditions, including necrotizing soft tissue infection and streptococcal toxic shock. In addition, the post-infectious syndrome of acute rheumatic fever and the long-term consequences of rheumatic heart disease remain prevalent in resource-limited countries and account for the majority of the estimated 500,000 deaths attributed to GAS infection annually (15, 16). The importance of SLO in the biology and pathogenesis of GAS infection is supported by the nearly universal production of SLO by clinical isolates, attenuation of SLO-deficient mutants in experimental infection models, and the molecular epidemiological observation that invasive clones of GAS that have emerged during and since the 1980s exhibit genomic evidence of acquisition of a highly active promoter driving increased expression of the operon encoding SLO and NADase (17, 18). These experimental and epidemiological observations, together with the immunogenicity of SLO during natural infection, have led to the inclusion of detoxified SLO as a component of multivalent GAS protein vaccines currently in preclinical testing (19–21).

To better understand how SLO interacts with host cells, we performed unbiased CRISPR-Cas9 screens to identify host factors that confer susceptibility to SLO cytotoxicity, focusing on the role of SLO binding to cell surface glycans in disease pathogenesis.

## RESULTS

### An oligosaccharide that binds SLO *in vitro* inhibits SLO binding to human epidermal epithelial cells

**Fig 1:**
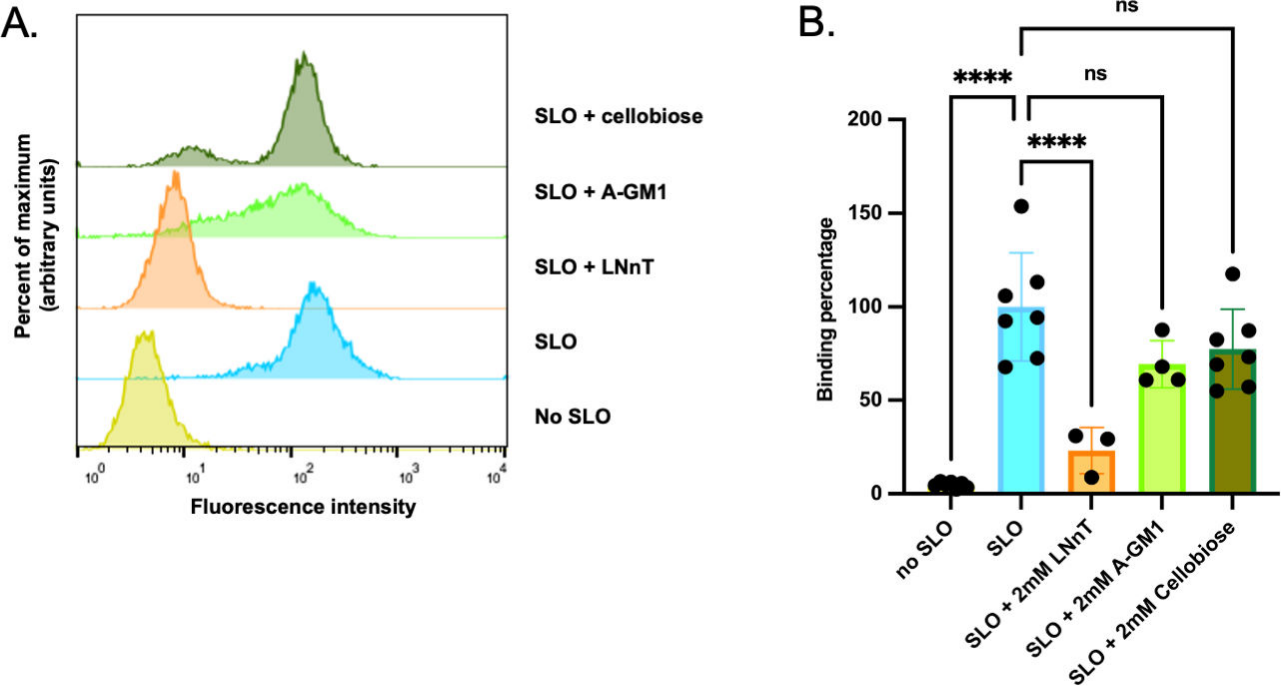
An oligosaccharide that binds SLO *in vitro* inhibits SLO binding to human epidermal cells. (**A**) Representative flow cytometry profile for the binding of SLO to A431 cells in the absence or presence of oligosaccharide inhibitor lacto-*N*-neotetraose (LNnt), ganglioside asialo GM1 head group (A-GM1), or cellobiose. (**B**) Quantification of mean fluorescence intensity values for ≥3 independent replicates of the experiment illustrated in panel A. The mean value for SLO in the absence of inhibitor is set at 100%. ****P < 0.0001.

Previous studies using glycan arrays and surface plasmon resonance found that SLO bound a variety of glycan structures, many of which contained a terminal galactose residue. SLO bound with high affinity to the human milk oligosaccharide lacto-*N*-neotetraose (LNnT, galβ1-4glcNAcβ1-3galβ1-4glc, *K*_*D*_ 0.93 nM). Furthermore, LNnT could inhibit SLO-mediated hemolysis of human erythrocytes, an observation that suggested cell surface glycans might participate in toxin action (13, 14). To investigate whether these findings could be extended to epithelial cell types typically colonized or infected by GAS to cause disease, we tested SLO binding to human epithelial A431 cells and if such binding could be inhibited by competition with LNnT. As predicted, using flow cytometry, we detected the binding of SLO to A431 cells in the absence of competing glycans, and SLO binding was efficiently blocked by the addition of 2 mM LNnT (Fig. 1). We also observed more modest and not statistically significant inhibition by the carbohydrate head group of ganglioside asialo-GM1 (A-GM1, galβ1-3galNAcβ1-4galβ1-4glc), which was also bound by SLO in glycan array experiments and by cellobiose (glcβ1-4glc) (14). The finding that a soluble oligosaccharide inhibitor can block the binding of SLO to human epidermal epithelial cells implies that cell surface glycans represent binding sites for SLO on epithelial surfaces colonized and infected by GAS.

### A CRISPR-Cas9 screen identifies glycosphingolipid biosynthetic enzymes as key host factors for SLO-mediated cytotoxicity

To understand how glycan binding may affect SLO toxicity, we developed a comprehensive and unbiased approach to search for host factors required for SLO binding to and the poration of host cells. Our screening strategy was based on CRISPR-Cas9 inactivation of genes required for susceptibility to SLO-mediated cytotoxicity using the near-haploid cell line HAP1, a cell line derived from the chronic myelocytic leukemia cell line KBM-7 (22). Except for the disomy of chromosome 8, each gene is represented only once, thus enhancing the power to detect a null phenotype by sgRNA inactivation of a genetic locus. Carette et al. (23) exploited the near-haploid gene content of this cell line to identify host factors for several pathogens using a loss-of-function insertional mutagenesis strategy, work that established proof of principle for this approach.

**Fig 2:**
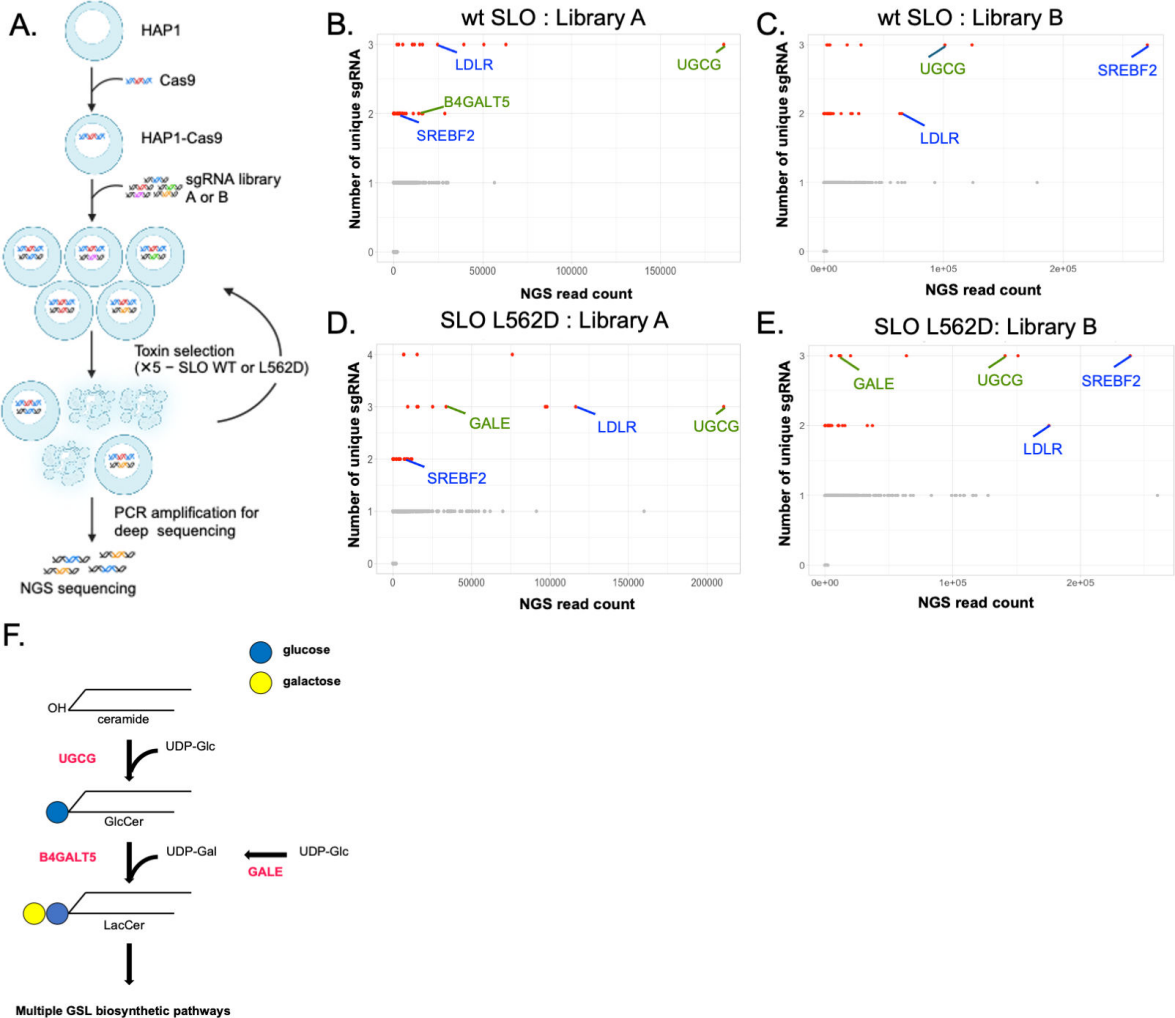
CRISPR-Cas9 screens identify glycosphingolipid biosynthetic enzymes as key host factors for SLO-mediated cytotoxicity. (**A**) Schematic of CRISPR screen workflow. (B–E) Results of four independent CRISPR screens in HAP1-Cas9 cells using native SLO (panels B and C) or SLO L562D (panels D and E). Each data point represents a single gene plotted as total sgRNA read counts on the *x*-axis versus number of unique sgRNA sequences on the *y*-axis. Selected genes involved in cholesterol homeostasis (blue) or glycosphingolipid biosynthesis (green) are indicated. See also Table 1. (**F**) Schematic of the earliest glycosylation steps in the biosynthesis of glucosyl glycosphingolipids. Enzymes encoded by genes identified in CRISPR-Cas9 screens for SLO susceptibility factors are labeled in red. UGCG, UDP-glucose ceramide glucosyltransferase; B4GALT5, β-1,4-galactosyltransferase 5; GALE, UDP-galactose-4-epimerase; GlcCer, glucosylceramide; and LacCer, lactosylceramide.

Preliminary experiments demonstrated that HAP1 cells were susceptible to SLO-mediated lysis, with ~90% cell death after exposure to ~5 nm SLO. Accordingly, we used the human CRISPR knockout GeCKO v2 two-plasmid system for a CRISPR-Cas9 screen of SLO cytotoxicity in HAP1 cells (24, 25). The GeCKO system consists of two single-guide RNA (sgRNA) libraries, A and B, each containing ~60,000 individual sgRNAs. The two libraries each contain three different sgRNAs targeting each of 19,050 human genes and 1,000 control non-targeting sgRNAs. After the introduction of Cas9 into HAP1 cells, we isolated and expanded individual cells and selected a high-expressing clone for expansion as it has been suggested that gene-editing activity is more robust in a high-expressing single cell clone compared to the bulk cell population (26). CRISPR libraries A and B were introduced separately into the Cas9 high-expressing clone for independent screens of each library. The HAP1-CRISPR-Cas9 libraries were expanded and then each was subjected to five sequential rounds of selection with purified recombinant SLO at increasing concentrations from 5 to 80 nm to achieve approximately 90% cell killing at each round (Fig. 2A).

To increase specificity for non-cholesterol receptors, we performed additional screens using an SLO variant that is defective for cholesterol binding. For this purpose, we replaced leucine 562 with aspartic acid in the cholesterol-binding site to produce SLO L562D (27). The mutant toxin was expressed as an N-terminal 6×His fusion and purified by Ni-affinity and gel filtration chromatography. In preliminary experiments, we found that the cytolytic activity of SLO L562D was markedly reduced compared to that of wild-type SLO, but not entirely absent. While wild-type SLO at a concentration of 5 nM resulted in ~90% cytotoxicity for HAP1 cells, L562D only achieved this degree of cytotoxicity at a concentration of 1 µM. We used the same HAP1-CRISPR-Cas9 libraries A and B to perform two additional screens using recombinant SLO L562D. Despite the reduced cytotoxic activity of L562D SLO, we found it was possible to perform four sequential rounds of selection, starting with an initial concentration of mutant toxin of 1 µM and doubling the concentration to achieve ~90% cell killing with each successive round.

For both the screens using native SLO and those using SLO L562D, deep sequencing of the starting cell population and cells from subsequent rounds of selection revealed progressive enrichment of sgRNAs targeting several genes of potential interest for interaction with SLO. Among the top hits were genes involved in the regulation of cholesterol metabolism and homeostasis. These genes included *ldlr* encoding low-density lipoprotein receptor, which binds low-density lipoprotein, the primary cholesterol-carrying lipoprotein in plasma, and transports it into cells, and *srebf2* encoding sterol regulatory element-binding transcription factor 2, an important regulator of cholesterol biosynthesis (28–30) (Fig. 2B through E; Table 1). As membrane cholesterol is known to be essential for SLO-pore formation, enrichment for genes encoding key proteins in cholesterol homeostasis validated the screens and their ability to detect host factors required for SLO cytotoxicity (1).

**TABLE 1:** Selected top hits from CRISPR-Cas9 screens in Hap1 cells for host factors mediating susceptibility to SLO or SLO L562D^*a*^

Gene	WT SLO				SLO L562D			
	No. sgRNAs enriched		Mean log_2_ fold change		No. sgRNAs enriched		Mean log_2_ fold change	
	LibA	LibB	LibA	LibB	LibA	LibB	LibA	LibB
Cholesterol regulation								
*ldlr*	3	2	4.3	3.1	3	3	6.1	5.1
*srebf2*	2	3	3.2	4.1	2	3	4.2	5.6
Glycosphingolipid biosynthesis								
*ugcg*	3	3	5.1	3.9	3	3	5.6	6.0
*b4galt5*	2	0	3.7	3.0	0	0	0.4	2.7
*gale*	0	1	2.7	2.6	3	3	5.3	5.8

^*a*^ Data represent the number of sgRNAs enriched in library A (LibA) or B (LibB) and mean log_2_ fold change in read counts in round 4 or 5 of selection over the starting population.

Also, among the most highly enriched genes were those encoding enzymes with functions in glycosphingolipid (GSL) biosynthesis: *ugcg* encoding UDP-glucose ceramide glucosyltransferase, which catalyzes the addition of glucose to ceramide, the first glycosylation step in the biosynthesis of glucosyl GSLs; *b4galt5* encoding β-1,4-galactosyltransferase 5, which catalyzes the transfer of galactose from UDP-gal to glucosylceramide, the subsequent reaction in the synthesis of several families of GSLs, including all gangliosides; and *gale* encoding UDP-galactose-4-epimerase to produce UDP-gal, which is a substrate for B4GALT5 (31, 32) (Fig. 2B through F; Table 1). These results, obtained concordantly in two independent CRISPR-Cas9 screens using two separate sgRNA libraries, implicate a role for host cell membrane GSLs in SLO-induced toxicity.

### Targeted inactivation of GSL biosynthetic genes confers resistance to SLO cytotoxicity

**Fig 3:**
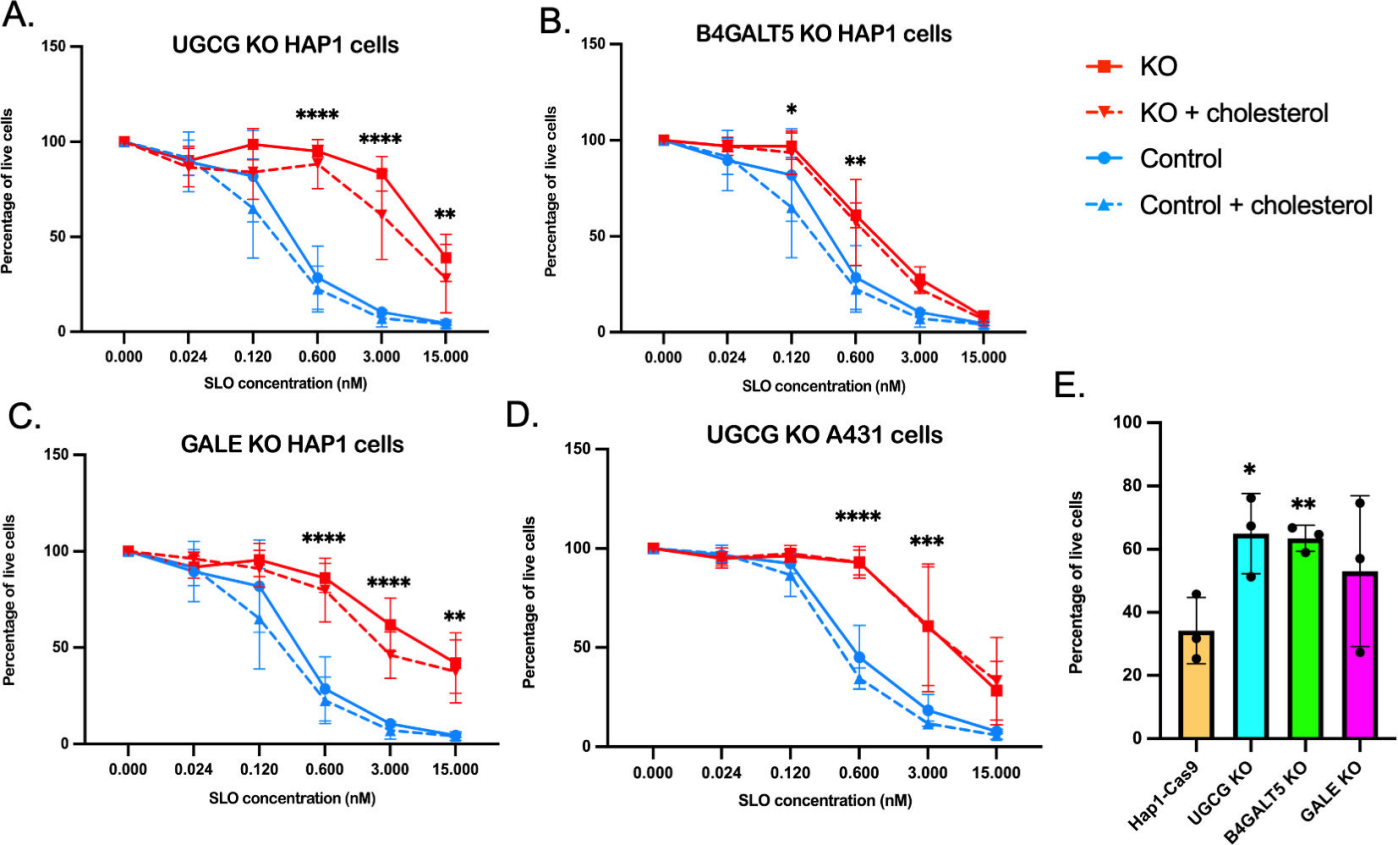
Targeted inactivation of glycosphingolipid biosynthetic genes confers resistance to SLO cytotoxicity. (A–C) Dose-response curves for SLO-mediated cytotoxicity of HAP1-Cas9 cell lines with targeted knockouts in individual genes encoding glycosphingolipid biosynthetic enzymes (red) compared to that for HAP1-Cas9 control cells (blue, repeated for clarity in each panel). (**D**) Dose-response curve for SLO-mediated cytotoxicity of a *ugcg* targeted knockout in A431 cells (red) compared to that for A431 control cells (blue). In panels A–D, dashed lines indicate results for cells treated with supplemental cholesterol prior to SLO exposure. (**E**) Resistance to SLO cytotoxicity of HAP1-Cas9 cell lines deficient in the expression of glycosphingolipid biosynthetic enzymes is recapitulated by challenge with live bacteria. Data represent mean ± SD percentage of surviving cells after exposure of cell monolayers to GAS strain 854. For all panels, data represent mean values ± SD for three independent experiments. *P < 0.05, **P < 0.01, and ****P < 0.0001 for comparison with control. Abbreviations are as in Fig. 2; KO, knockout.

To validate these gene hits, we constructed HAP1 cell lines in which we inactivated each of the genes encoding the early enzymes in GSL biosynthesis: *ugcg, b4galt5,* and *gale*. For each of the three candidate genes, we introduced a CRISPR knockout construct, which harbors the sgRNA sequence that gave the highest read count in one or both screens, into HAP1-Cas9 cells. DNA sequencing confirmed the disruption of the targeted coding sequence in each cell line. We tested the susceptibility of each of the knockout (KO) cell lines to SLO cytotoxicity and found that the SLO concentration required to achieve 50% lysis of each of the mutant cell lines was shifted ~3- to 30-fold higher than that for control HAP1-Cas9 cells (Fig. 3A through C). The increase in resistance to SLO cytotoxicity was somewhat lower in the *b4galt5* KO compared to the *ugcg* and *gale* KOs, perhaps reflecting the fact that the reaction catalyzed by B4GALT5 can also be mediated by β-4-galactosyltransferase 6, which may partially compensate for the absence of B4GALT5. These results strongly support the involvement of GSLs in SLO-mediated cell toxicity and are compatible with such structures participating in SLO binding to the cell surface. We then performed the same experiment in A431 cells containing the *ugcg* deletion (33) to test if these results can be generalized to epithelial cell types. Like the GSL biosynthesis enzyme KOs in HAP1 cells, the *ugcg* mutant in A431 epithelial cells exhibited a log-fold reduced susceptibility to SLO-mediated cytotoxicity compared to wild-type A431 cells (Fig. 3D).

To confirm and extend these results, we tested the susceptibility of the mutant HAP1 cell lines to cytotoxic injury during *in vitro* infection by the wild-type GAS strain 854. This strain produces both SLO and enzymatically active NADase, which acts synergistically with SLO to enhance cytotoxicity (6, 7, 34). Consistent with results from experiments using purified SLO, we observed increased resistance to cell lysis/death during GAS infection of all three mutant cell lines compared to control HAP1-Cas9 cells, a difference that reached statistical significance for both *ugcg* and *b4galt5* KOs (Fig. 3E). These results implicate a mechanistic role for GSLs in SLO-mediated cell toxicity, not only during exposure to SLO in solution but also when host cells are exposed to an SLO- and NADase-producing GAS strain, as occurs during mucosal infection.

### Resistance of GSL mutants to SLO-toxicity cannot be fully explained by gene deletion effects on membrane cholesterol or toxin binding

**Fig 4:**
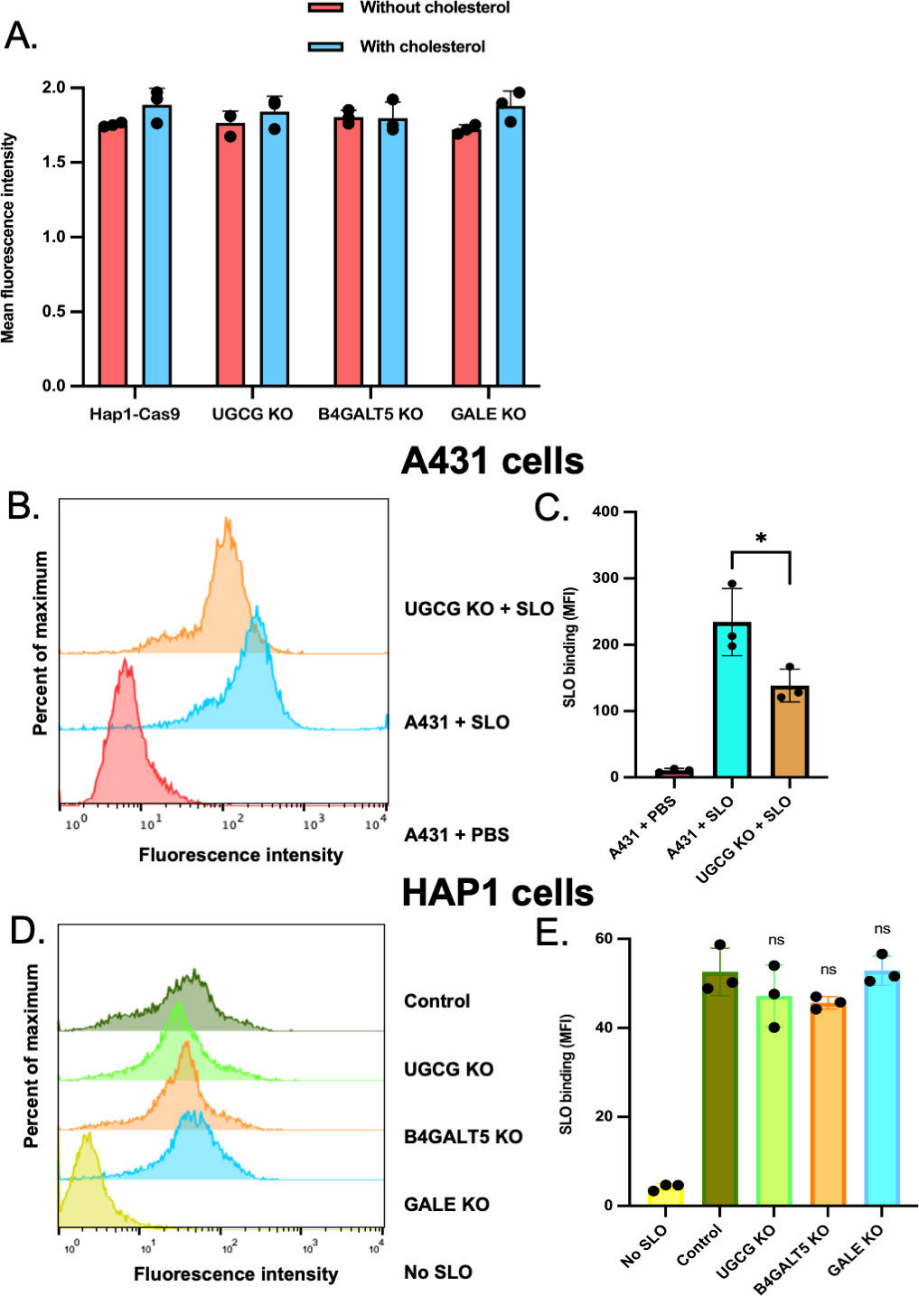
Resistance of GSL mutants to SLO toxicity cannot be fully explained by gene deletion effects on membrane cholesterol or toxin binding. (**A**) Plasma membrane cholesterol is not significantly reduced in HAP1-Cas9 cell lines deficient in the expression of glycosphingolipid biosynthetic enzymes. Each data point represents mean fluorescence intensity of filipin staining of the cell surface from at least 25 cells per slide on ≥3 slides per cell sample without (red bars) or with (blue bars) the addition of supplemental cholesterol. (**B**) Representative flow cytometry profile of SLO binding to wild-type A431 cells or *ugcg* knockout A431 cells. (**C**) Quantification of mean ± SD fluorescence intensity values for three independent replicates of the experiment illustrated in panel B. (**D**) Representative flow cytometry profiles for the binding of SLO to HAP1-Cas9 control cells or HAP1-Cas9 cell lines with targeted knockouts in individual genes encoding glycosphingolipid biosynthetic enzyme. (**E**) Quantification of mean fluorescence intensity values for three independent replicates of the experiment illustrated in panel D. Abbreviations are as in Fig. 2 and 3.

In eukaryotic cells, GSLs and cholesterol assemble together to affect membrane structure and function (31–33), and this interaction between GSLs and cholesterol could explain the resistance to SLO-mediated cytotoxicity observed in our functional studies of GSL KOs (Fig. 3). Accordingly, we first measured membrane cholesterol content in mutant cell lines lacking GSL expression. Total membrane cholesterol was quantified by measuring the fluorescence induced by the cholesterol-binding agent filipin. These studies revealed no significant difference between wild-type and mutant cells (Fig. 4A), implying that cholesterol was not rate limiting for SLO-induced toxicity in host cells lacking the GSLs. To confirm this interpretation, we introduced supplemental cholesterol into the plasma membrane of HAP1 or A431 cells and their respective GSL KO mutants by incubating them with cholesterol complexed with methyl-β-cyclodextrin. The relative resistance of the KO cells compared to the wild type was not changed (dashed lines in Fig. 3A through D).

We next tested toxin binding. When quantified by flow cytometry analysis, SLO binding to the *ugcg* KO in A431 cells was modestly reduced (~40%) compared to that for wild-type A431 cells (Fig. 4B and C), but SLO binding to each of the mutant HAP1-Cas9 cells lacking GSL expression, compared to HAP1-Cas9 parent cells, was not affected (Fig. 4D and E). Thus, while loss of GSL expression caused a small reduction in SLO binding to the cell surface of A431 cells (less than twofold), this was not observed in HAP1 cells, and neither result can explain the >10-fold reduction in cytotoxicity observed in these mutant cell lines. Such results are biologically plausible as cell surface GSLs represent only a subset of potential glycan receptors for SLO. Toxin binding to other glycans abundantly expressed on the cell surface of eukaryotic cells likely compensates for the absence of GSLs with respect to overall binding.

### Binding to GSLs targets SLO to cholesterol-rich lipid rafts

GSLs are not distributed uniformly over the cell surface. Rather, they are clustered in membrane nanodomains or lipid rafts, which are also enriched for cholesterol (31, 32). Moreover, the close apposition of cholesterol molecules alongside the ceramide moiety of the complex sphingolipids (sphingomyelin as well as the glycosphingolipids studied here) enables the assembly of these cholesterol-rich membrane nanodomains (33, 35). Since the reduced susceptibility of GSL KO cells was not convincingly explained by a reduction in overall binding of SLO or by a reduction in plasma membrane cholesterol content, we hypothesized that SLO binding to GSLs, but not to other glycan receptors, may target SLO to cholesterol-rich lipid rafts. If that were the case, loss of GSLs might not reduce overall SLO binding to the cell but rather reduce the GSL-mediated localization of SLO to cholesterol-rich membrane domains that are most susceptible to SLO-pore formation.

**Fig 5:**
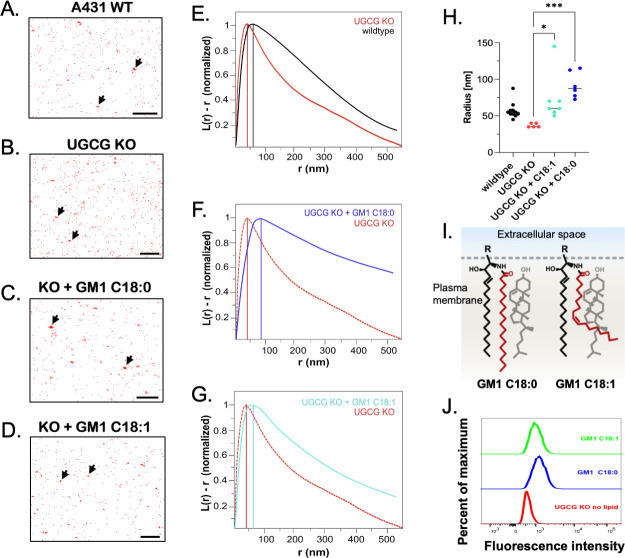
Reconstitution of glycosphingolipid-deficient A431 cells with exogenous GM1 ganglioside increases SLO clustering on the cell surface. (A–D) Representative STORM images of SLO bound to the surface of wild-type A431 cells, the *ugcg* knockout, and the knockout supplemented with ganglioside GM1 C18:0 or with GM1 18:1 (**A, B, C, and D**, respectively). Cell monolayers were exposed to (non-hemolytic) SLO G395V G396V labeled with Alexa Fluor 647 and then imaged by STORM. Typical clusters of bound SLO are indicated by black arrows for each condition. Scale bar is 500 nm. (E–G) Clustering analysis of SLO binding to wild-type A431 cells or to *ugcg* knockout cells without or with supplemental ganglioside GM1. Representative curves from five independent replicates of the experiments illustrated in panels A–D are plotted according to Ripley’s K-function analysis of clustering of labeled SLO molecules into domains of radius *r* on the *x*-axis versus *L*(*r*) – *r* on the *y*-axis, in which for a given radius *r*, *L*(*r*) is the radius within which the number of points would be distributed if the distribution were completely random. Thus, the maximum of each curve corresponds to the cluster diameter containing the highest density of SLO molecules. (**E**) Vertical lines mark the maximum of curves corresponding to SLO binding to wild-type A431 cells (black) or *ugcg* knockout cells (red). (**F and G**) A vertical line marks the maximum of a curve corresponding to SLO binding to *ugcg* KO cells supplemented with ganglioside GM1 18:0 (panel F, blue) or GM1 18:1 (panel G, cyan). The curve from panel E for unsupplemented *ugcg* knockout cells is included for comparison (red). (**H**) Summary data for experiments illustrated in panels E–G. Statistically significant differences between groups are indicated. *P < 0.05 and ***P < 0.001. (**I**) Schematic of ganglioside GM1 with a fully saturated fatty acyl chain (C18:0) or with a single double bond at position 9 (C18:1). The GM1 oligosaccharide head group is represented by “R” (drawing modified from reference 33). An adjacent cholesterol molecule (shown in gray) packs efficiently with native GM1 C18:0 but not with GM1 C18:1 due to the bend in its acyl chain. (**J**) For SLO clustering experiments in panels F and G, glycosphingolipid-deficient cells were loaded with 5 µM GM1 C18:0 (blue) or 0.5 µM GM1 C18:1 (green), which resulted in similar levels of cell surface GM1 expression. Data represent mean fluorescence intensity as assessed by flow cytometry after staining cells with cholera toxin B-Alexa Fluor 488, which binds to ganglioside GM1.

To investigate the distribution of SLO molecules bound to human epidermal epithelial cells, we introduced two point mutations in domain 3 of SLO to reduce cytotoxicity (hereafter, non-hemolytic or nhSLO). These mutations, G395V, G396V, have been shown previously to prevent SLO oligomerization and pore formation but not to interfere with SLO binding to host cells (10, 36). We exposed the apical surface of a monolayer of *ugcg* KO or wild-type A431 cells to nhSLO labeled with Alexa Fluor 647. After washing, the cells were imaged by stochastic optical reconstruction microscopy (STORM). Image analysis suggested greater clustering of SLO on wild-type cells compared to the *ugcg* KO, although the difference did not reach statistical significance. This result is consistent with the hypothesis that SLO binding is concentrated at the foci of GSLs on the plasma membrane (Fig. 5A, B, and E).

To test this idea, we used GM1 glycosphingolipids synthesized to contain ceramide moieties with or without the motif that enables close assembly with cholesterol and nanodomain formation (33). SLO has been shown to bind to the asialo GM1 oligosaccharide head group *in vitro* (14). We found that cell surface clustering of SLO on mutant *ugcg* KO A431 cells was markedly increased by reconstitution with the exogenously applied GM1 ceramide C18:0 species that can assemble closely with cholesterol to form raft nanodomains (Fig. 5C, F, and H). We then tested the GM1 variant, GM1 C18:1^Δ9^, in which the 18-carbon saturated fatty acid tail is interrupted at position 9 by a single double bond. The double bond introduces a bend or kink in the lipid tail that prevents efficient interaction of the ganglioside lipid chain with membrane cholesterol and assembly in raft microdomains (33, 37). Whereas the linear structure of the native GM1 fully saturated (C18:0) fatty acid acyl chain packs efficiently with adjacent cholesterol molecules, such intermolecular packing interaction is blocked by the bent structure of the variant GM1 C18:1^Δ9^ (Fig. 5I). We found that reconstitution with the GM1 ceramide C18:1^Δ9^ species was less effective than GM1 18:0 in increasing SLO clustering (Fig. 5D, G, and H). These results are consistent with the binding of SLO to GSLs assembled in cholesterol-rich membrane nanodomains.

**Fig 6:**
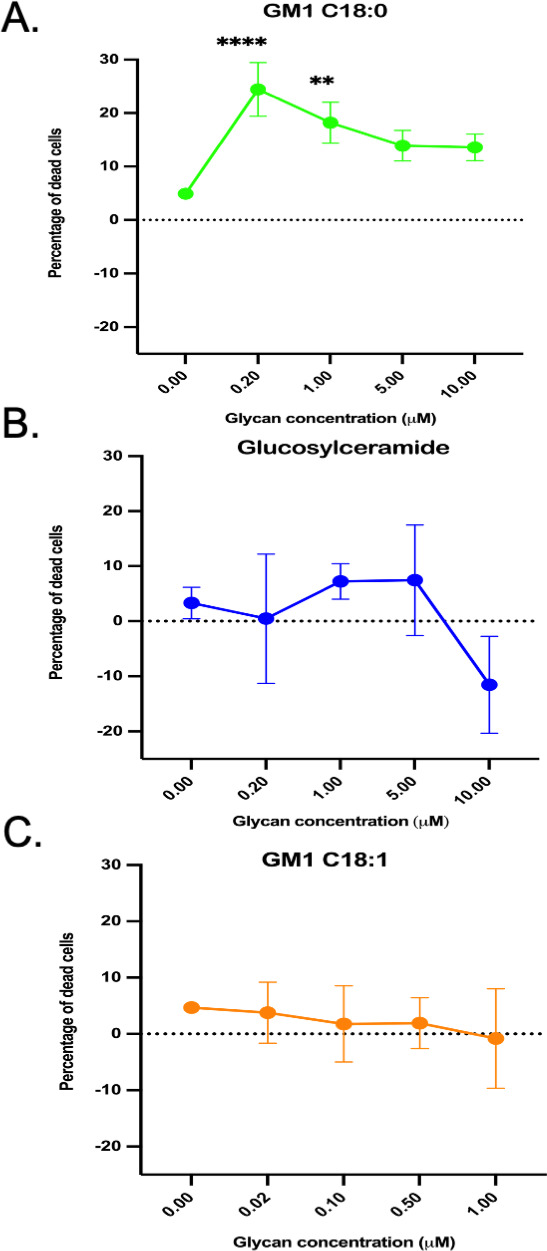
Reconstitution of glycosphingolipid-deficient A431 cells with exogenous GM1 ganglioside restores susceptibility to SLO cytotoxicity. A431 *ugcg* knockout cell monolayers were treated with the indicated concentration of glycan prior to exposure to 0.6 nM SLO. Glycans are (**A**) ganglioside GM1 C18:0, (**B**) glucosylceramide, or (**C**) ganglioside GM1 C18:1. **P < 0.01 and ****P < 0.0001 for comparison to no added glycan.

We extended this structural analysis by testing directly for toxin function. We examined whether reconstitution of *ugcg* KO cells with the different GM1 species could restore the cell’s susceptibility to SLO-mediated cytotoxicity. Incorporation of the GM1 C18:1^Δ9^ variant into *ugcg* KO cells resulted in a similar level of enhanced surface expression compared to the GM1 C18:0 variant, as assessed by binding of cholera toxin B subunit (CTX-B) (Fig. 5J). However, only GM1 C18:0 and not the C18:1^Δ9^ variant restored SLO susceptibility (Fig. 6A and C). This finding is consistent with the hypothesis that the physical proximity of GSLs to membrane cholesterol by assembly in membrane nanodomains enhances SLO pore-forming activity.

Finally, to test if the rescue of SLO cytotoxicity required binding directly to GM1 reconstituted in the membrane, we tested *ugcg* KO cells reconstituted with glucosylceramide. This glycosphingolipid contains a ceramide domain containing a C18:0 acyl chain predicted to assemble closely with cholesterol in membrane nanodomains like the GM1 C18:0 variant, but it lacks the glycan moiety that can bind SLO. We found that the incorporation of exogenous glucosylceramide failed to rescue SLO-induced cytotoxicity (Fig. 6B), indicating the necessity for toxin binding directly to the GSL.

The results of these structural studies support the hypothesis that SLO binding to GSLs enhances pore formation and cytotoxicity by recruiting SLO to membrane nanodomains rich in cholesterol.

## DISCUSSION

We found that cell surface glycosphingolipids can serve as complementary binding sites for SLO on epithelial cells that target the toxin to cholesterol-rich membrane nanodomains for enhanced cytotoxicity. As such, glycosphingolipids represent one of the major host cell susceptibility factors, alongside membrane cholesterol, for cytotoxic injury associated with GAS infection.

Earlier studies indicated that oligosaccharides of diverse structures bind to SLO *in vitro* (13, 14). A terminal galactose residue is present on many but not all identified SLO-binding oligosaccharides, and conservation of more extensive structural motifs is not evident. The diversity of recognized glycans suggests that SLO binding can be promiscuous, with multiple cell surface glycans serving as potential binding sites—though not all are functionally relevant to toxin action, as we found here.

Using unbiased CRISPR screens, we identified enzymes early in the biosynthetic pathway of GSLs as host susceptibility factors for SLO cytotoxicity. Construction and testing of cell lines harboring targeted knockouts of the genes encoding these enzymes confirmed their importance in susceptibility to SLO-mediated cytotoxicity. That the identified enzymes act at the earliest steps in glycosphingolipid synthesis and not on more specialized downstream pathways is compatible with *in vitro* observations that SLO can bind a variety of oligosaccharide motifs. Rather than pointing to a specific glycan structure, our results imply that a particular class of cell surface GSLs, those containing glycans capable of binding SLO and with a ceramide domain capable of assembling closely with cholesterol in membrane nanodomains, are the functionally relevant glycan targets for SLO pore formation.

The finding that GSL deficiency had a minimal effect on the overall binding of SLO to the cell surface suggests a more specific functional role for SLO binding to GSLs in enhancing pore formation compared to binding to other glycosylated cell surface molecules. GSLs are not distributed uniformly throughout the plasma membrane. Most GSLs contain ceramide domains with saturated acyl chains, and these are concentrated in membrane nanodomains or lipid rafts rich in cholesterol (31, 37). Binding to GSLs concentrates SLO within cholesterol-rich lipid rafts, thus facilitating SLO binding to adjacent cholesterol molecules, oligomerization, and insertion into the cell membrane. In a similar fashion, CD59, the glycosylphosphatidylinositol-linked receptor for intermedilysin, is also enriched in lipid rafts, and binding of intermedilysin to this non-cholesterol receptor also concentrates bound toxin in membrane nanodomains rich in cholesterol required for pore formation (38, 39).

SLO is secreted into the extracellular space, so it is possible for monomers in solution to encounter and bind to nearby cells through passive diffusion. However, during infection, GAS adheres to mucosal epithelial cells. Secretion of SLO and its co-toxin NADase from adherent bacteria increases the local concentration of both toxins and markedly increases the efficiency of SLO-mediated translocation of NADase into host cells (7). Integrins displayed on host cells are thought to play an important role in GAS attachment by binding plasma proteins such as fibrinogen and fibronectin, which serve as bridging molecules that bind to GAS surface proteins including M protein and fibronectin-binding proteins (40, 41). Another mechanism of GAS binding is the interaction of the hyaluronic acid capsular polysaccharide with the hyaluronic acid-binding protein CD44, which is found on the surface of epithelial and hematopoietic cells (42, 43). Both integrins and CD44 are concentrated in lipid rafts (44–46). Crosslinking of these raft-associated proteins by binding to multiple sites on the bacterial surface could promote coalescence of individual rafts into larger domains, increasing local enrichment of GSLs and cholesterol in the plasma membrane and further enhancing the efficiency of SLO binding and pore formation.

The current study adds a new dimension to the hypothesis that cell surface glycans serve as co-receptors for CDCs along with plasma membrane cholesterol. Our unbiased genetic screens identified GSL biosynthetic genes as key host susceptibility factors for SLO cytotoxicity. Characterization of KO cell lines and reconstitution with structurally defined GSLs provide strong evidence that GSLs play a critical role in recruiting SLO to lipid rafts. GSL-bound SLO is optimally positioned to bind adjacent cholesterol molecules, which are enriched in rafts, thereby promoting SLO-mediated pore formation and cytotoxic injury to host cells. In this way, recognition of cell surface GSLs enhances the effectiveness of SLO as a pore-forming toxin and virulence factor in GAS infection.

## MATERIALS AND METHODS

See File S1 for additional details.

### Bacterial strains

GAS strain 854 is an M type 1 clinical isolate from a patient with a retroperitoneal abscess (47, 48). *Escherichia coli* strain NEB5α (New England Biolabs) was used for plasmid manipulation, and strain BL21 (New England Biolabs) was used for protein expression.

### Cell culture

HAP1 is a near-haploid cell line derived from the human chronic myelocytic leukemia line KBM7 (Horizon Discovery). A431 is a human epidermoid carcinoma cell line (ATCC); A431*ugcg* is a mutant in which *ugcg* has been deleted (33).

### Cloning and mutagenesis/DNA manipulation

An expression plasmid for the production of SLO L562D was constructed by QuikChange site-directed mutagenesis according to the manufacturer’s recommendations (Agilent Technologies) using pETslo as template for amplification (27).

### Protein expression and purification

Expression and purification of native SLO were described previously (28). SLO L562D was expressed and purified as described previously for native SLO, with minor modifications.

### SLO binding and binding inhibition assays

Cells were grown to 70%–80% confluence, detached with 0.25% trypsin, washed and suspended in PBS, and distributed in 96-well plates at a density of 5 × 10^5^ cells per well. Cells were incubated in PBS with 1 mM DTT and 25 nM SLO at 4°C for 30 min. (For binding inhibition experiments, SLO was pre-incubated with 2 mM oligosaccharide at 37°C for 30 min before addition to cells.) SLO was removed, and the cells were washed twice with PBS. Cells were incubated at 4°C for 1 hour with rabbit anti-SLO IgG (34) at 1:50 dilution in 1% BSA in PBS and then washed twice with PBS. Alexa Fluor 488 donkey anti-rabbit IgG (H + L, Invitrogen by ThermoFisher Scientific) at 1:500 dilution in 1% BSA in PBS was added and incubated at 4°C for 30 min. The cells were washed twice with PBS, fixed in 2% paraformaldehyde at 4°C for 15 min in the dark, washed twice with PBS, and resuspended at a final volume of 200 µL in PBS. Prepared cells were subjected to flow cytometry (Becton Dickinson FACSCalibur), and data were analyzed using FlowJo software. Statistical analysis was performed using GraphPad Prism9.

### CRISPR screen for SLO susceptibility factors

Genome-scale CRISPR screening was performed in HAP1 cells using the human CRISPR knockout GeCKO version 2.0 two-vector system (25). Screening was done as described previously with modifications as described in File S1 (49).

### Bioinformatic analysis of NGS screening data

Data analysis was performed using Cutadapt/1.14, Bowtie2/2.3.4.3, and MAGeCK/0.5.9.4 (50–52). R studio ggplot was used for the visualization of gene enrichment and the number of sgRNAs enriched for each gene (53).

### Construction of HAP1 cell lines harboring inactivating mutations of individual GSL biosynthesis genes

Mutant cell lines harboring inactivating mutations in individual GSL biosynthesis genes were constructed as described (49). Oligonucleotides used in the study to produce knockout constructs are listed in Table 2.

**TABLE 2:** Oligonucleotides used to create knockouts of individual GSL genes in Hap1 cells

Gene	DNA sequence
*gale*	5′ CACCGCCGGGATTACATCCATGTCG 3′
	5′ AAACCGACATGGATGTAATCCCGGC 3′
*ugcg*	5′ CACCGGCCTTACGTAGCAGACAGAC 3′
	5′ AAACGTCTGTCTGCTACGTAAGGC 3′
*b4galt5*	5′ CACCGGATCGCAACTATTATGGATG 3′
	5′ AAACCATCCATAATAGTTGCGATC 3′

### CellTiter-Glo cytotoxicity assay

The susceptibility of eukaryotic cells to SLO was assessed using CellTiter-Glo 2.0 Cell Viability Assay (Promega) according to the manufacturer’s recommendations.

### Augmentation of cell membrane cholesterol by incorporation of exogenous cholesterol

For some experiments, cell membrane cholesterol was augmented *in vitro* by the addition of water-soluble cholesterol complexed with methyl-β-cyclodextrin to a concentration of 120 µg/mL and incubated for 15 min at 37°C, after which the cells were washed with cell culture medium.

### Cell infection assay

GAS strain 854 was grown to the mid-exponential phase, collected by centrifugation, suspended in DMEM, and inoculated at a multiplicity of infection of 0.2 onto the surface of confluent HAP1-Cas9 knockout or control cells in 24-well plates. Infected cells were incubated at 37°C in 5% CO_2_ for 2 hours. Cells were washed twice with PBS and then incubated in DMEM with 1% FBS and 20 µg/mL penicillin overnight at 37°C in 5% CO_2_. Cell viability was determined 20–22 hours post-infection by trypan blue exclusion (54).

### Protein labeling with Alexa Fluor 647

Expression and purification of SLO G395V G396V were described previously (27, 55). Purified protein was labeled with Alexa Fluor 647 according to the manufacturer’s recommendations (Alexa Fluor 647 NHS ester, Invitrogen).

### Reconstitution of membrane glycosphingolipids

A431 *ugcg* KO cells were seeded in opaque-walled 96-well plates at a density of 10,000 cells per well. Cells were allowed to adhere for 20 hours in DMEM at 37°C in 5% CO_2_. Cells were washed three times with prewarmed DMEM without serum. Individual glycosphingolipids were suspended at a range of concentrations in DMEM containing 5 µM defatted-bovine serum albumin (dfBSA; Sigma). Glycosphingolipids were equilibrated for 5 min at 37°C and then added to the cells and incubated at 37°C in 5% CO_2_ for 30 min. Cells were washed twice with 0.2 µM dfBSA in DMEM and then tested for susceptibility to SLO-mediated cytotoxicity by incubation with 0.6 nM SLO, as described above.

### Incorporation of GM1 analogs and SLO binding

GM1 C18:0 or GM1 C18:1^Δ9^ complexed to dfBSA in a 1:0.75 ratio were added to A431 *ugcg* KO cells at 5 or 0.5 µM, respectively. Equal incorporation of GM1 C18:0 and GM1 C18:1^Δ9^ was validated by FACS using fluorescently labeled cholera toxin B-subunit. For SLO binding, SLO G395V G396V-Alexa Fluor 647 was added at 20–200 nM for 15 min at 4°C. Cells were fixed after SLO addition using 0.2% glutaraldehyde and 4% formaldehyde and then imaged by STORM (37).

### Super-resolution microscopy

Super-resolution microscopy was performed using an ELYRA7 and 8 microscope. See also File S1.

### Statistical analysis

Statistical significance of differences between groups was evaluated using one-way ANOVA with Tukey’s or Dunnett’s multiple comparison test (SLO binding and inhibition assays, SLO clustering analysis), two-way ANOVA with Tukey’s or Dunnett’s multiple comparison test (SLO cytotoxicity assays), or one sample *t* test and Wilcoxon test (GAS infection assays).

## SUPPLEMENTAL MATERIAL

Table S1 mbio.03777-24-s0001.xlsx CRISPR library A.

Table S2 mbio.03777-24-s0002.xlsx CRISPR library B.

File S1 mbio.03777-24-s0003.docx Additional information.

ASM does not own the copyrights to Supplemental Material that may be linked to, or accessed through, an article. The authors have granted ASM a non-exclusive, world-wide license to publish the Supplemental Material files. Please contact the corresponding author directly for reuse.
