# Supplementary material for: SLO co-opts host cell glycosphingolipids to access cholesterol-rich lipid rafts for enhanced pore formation and cytotoxicity
Source: mBio. 2025 Jan 21;16(3):e03777-24. doi: 10.1128/mbio.03777-24 (PMC11898750; doi:10.1128/mbio.03777-24)
Supplement: File S1 — Additional information. [file mbio.03777-24-s0003.docx]

# File S1

**Bacterial strains**

GAS strain 854 is an M type 1 clinical isolate from a patient with a retroperitoneal abscess (1, 2). GAS was grown at 37° C in Todd-Hewitt broth supplemented with 0.5% yeast extract. *E. coli* strain NEB5α (New England Biolabs) was used for plasmid manipulation and strain BL21 (New England Biolabs) was used for protein expression. *E. coli* was grown in Luria Bertani medium at 37° C unless otherwise indicated; when required, kanamycin was added at 50 μg/ml.

# Cell culture

HAP1 is a near haploid cell line derived from the human chronic myelocytic leukemia line KBM7 (Horizon Discovery). HAP1 cells were grown in IMDM medium (Gibco) supplemented with 10% fetal bovine serum (FBS). A431 is a human epidermoid carcinoma cell line (ATCC); A431*ugcg* is a mutant in which *ugcg* has been deleted (3). A431 cells were grown in DMEM (Gibco) with 10% FBS and 1% penicillin and streptomycin (HyClone). Both cell lines were maintained at 37° C in 5% CO2. During CRISPR-Cas9 screening and selection, HAP1 cells were maintained with 10

μg/ml blasticidin (InvivoGen) and 5 μg/ml puromycin (Gibco).

# Cloning and mutagenesis/DNA manipulation

An expression plasmid for production of SLO L562D was constructed by QuikChange site directed mutagenesis according to the manufacturer's recommendations (Agilent Technologies) using pETslo as template for amplification (4). Forward primer 5’ CTCAGGATCAACCGATAGCCCATATGG 3’ and reverse primer 5’ CCATATGGGCTATCGGTTGATCCTGAG 3’ were used to introduce a point mutation from leucine to aspartic acid at position 562 in the *slo* sequence. Fidelity of the resultant construct, named pETsloL562D, was confirmed by plasmid isolation and sequencing (Genewiz).

# Protein expression and purification

Expression and purification of native SLO was described previously (28). For expression of SLO L562D, pETsloL562D was transformed into *E. coli* BL21(DE3) competent cells. Protein expression was induced with IPTG during growth of selected transformants, as described previously for native SLO, with minor modification: cells were grown at 37° C to A600 ~ 0.3 and then induced with 0.8 mM IPTG at 30° C for 3 hrs. Cells were collected by centrifugation, lysed by sonication, and the lysate was purified by nickel affinity and gel filtration chromatography, as described (4). Purified SLO L562D was flash frozen in liquid nitrogen and stored at -80° C.

# SLO binding and binding-inhibition assays

Cells were grown to 70-80% confluence, detached with 0.25% trypsin, washed and suspended in PBS, and distributed in 96-well plates at a density of 5x10^5^ cells per well. Cells were incubated in PBS with 1 mM DTT and 25 nM SLO at 4° C for 30 min. (For binding inhibition experiments, SLO was pre-incubated with 2 mM glycan inhibitor at 37° C for 30 min before addition to cells.) SLO was removed, and the cells were washed twice with PBS. Cells were incubated at 4° C for 1 hr with rabbit anti-SLO IgG (5) at 1:50 dilution in 1% BSA in PBS and then washed twice with PBS. Alexa Fluor 488 donkey anti-rabbit IgG (H+L, Invitrogen by ThermoFisher Scientific) at 1:500 dilution in 1% BSA in PBS was added and incubated at 4° C for 30 minutes. The cells were washed twice with PBS, fixed in 2% paraformaldehyde at 4° C for 15 minutes in the dark, washed twice with PBS, resuspended at a final volume of 200 μl in PBS. Prepared cells were subjected to flow cytometry (Becton Dickinson FACSCalibur), and data were analyzed using FlowJo software. Statistical analysis was performed using GraphPad Prism9.

# CRISPR screen for SLO susceptibility factors

Genome scale CRISPR screening was performed in HAP1 cells using the human CRISPR knockout GeCKO v2.0 two-vector system (6). Screening was done as described previously with modifications as described below (7). HAP1 cells stably expressing Cas9 were transduced with either GeCKO v2 library A or library B (Human GeCKO v2 sgRNA library, Addgene, #1000000049) in LentiGuide-Puro plasmid (Addgene, #52963) for independent screens of library A and B. Two toxins—native (wt) SLO and SLO L562D—were used for separate screens of both libraries. HAP1-Cas9 cells were incubated in parallel with 1 mM DTT and either 5 nM wtSLO or 1

μM SLO L562D at 37° C in 5% CO2 for 24 hrs and then washed twice with PBS. Cells were released with trypsin, a portion was stored at -80°C for DNA sequencing, and the remaining cells were expanded in new dishes for a subsequent round of selection with a 2-fold higher toxin concentration. This procedure was repeated for five rounds of selection for wtSLO or four rounds for SLO L562D. Cells stored after each round of selection were used for isolation of genomic DNA. Sequences encoding single guide RNAs (sgRNA) were amplified using forward primer LentiGP-1_F 5’AATGGACTATCATATGCTTACCGTAACTTGAAAGTATTTCG3’ and reverse primer LentiGP-3_R 5’ATGAATACTGCCATTTGTCTCAAGATCTAGTTACGC3’ from each

genomic DNA sample and sent for Illumina NGS sequencing (Genewiz).

# Bioinformatic analysis of NGS screening data

Data analysis was performed on the O2 computing platform at Harvard Medical School. Cutadapt/1.14 software was used to trim adapter sequences (8); Bowtie2/2.3.4.3 software was used to align trimmed sequences with the GeCKO v2 human CRISPR knockout pooled library

(9); MAGeCK/0.5.9.4 software was used to identify enriched genes from the genome scale screening (10). R studio ggplot was used for visualization of gene enrichment and number of sgRNAs enriched for each gene (11).

# Construction of HAP1 cell lines harboring inactivating mutations of individual GSL biosynthesis genes.

Mutant cell lines harboring inactivating mutations in individual GSL biosynthesis genes were constructed as described ^48^. Oligonucleotides used in the study to produce knockout constructs are listed in **Table 2**. Briefly, oligonucleotides for *ugcg*, *b4galt5*, or *gale* were cloned into lentivirus vector and transformed into competent *E. coli* cells (NEB Stable (High Efficiency), NEB). Plasmids pMD2.G (envelope plasmid, Addgene, #12259) and psPAX2 (packaging plasmid, Addgene, #12260) were transformed in *E. coli* DH5α competent cells, as described (7). Transformants were confirmed by sequencing (Genewiz), and plasmid was prepared from confirmed clones (QIAprep Spin Miniprep kit, Qiagen).

Validated HAP1 cell lines were generated as described previously with the following modifications

(7). Lentivirus was used for transfection in HEK293T cells (Polyjet In Vitro DNA Transfection Reagent, SignaGen) and subsequent transduction into HAP1 cells for each of the three GSL biosynthetic genes. Genomic DNA was isolated from mutated cell lines (Blood & Cell Culture DNA Mini Kit, Qiagen), and fidelity of transduction was confirmed by sequencing (Genewiz).

# CellTiter-Glo cytotoxicity assay

Susceptibility of eukaryotic cells to SLO was assessed using CellTiter-Glo 2.0 Cell Viability Assay (Promega) according to the manufacturer’s recommendations. Opaque-walled 96-well plates were seeded with 10^4^ mammalian cells per well in cell-culture medium and incubated overnight at 37° C in 5% CO2. Cells were incubated in PBS containing 1 mM DTT and a range of SLO concentrations at 37° C in 5% CO2 for 30 minutes and then at room temperature for 30 minutes. An equal volume of CellTiter-Glo 2.0 Reagent was added (100 μl per well for 96-well plate). After mixing on an orbital shaker for 2 minutes to ensure cell lysis, samples were incubated at room

temperature for 10 minutes to stabilize the luminescence signal. Luminescence was recorded using a BioTek Synergy 2 plate reader.

**Augmentation of cell membrane cholesterol by incorporation of exogenous cholesterol** For some experiments, cell membrane cholesterol was augmented in vitro by addition of exogenous water-soluble cholesterol. For this purpose, water-soluble cholesterol complexed with methyl-β-cyclodextrin (MβCD, Sigma Aldrich) was added in cell culture medium to a concentration of 120 μg/mL and incubated for 15 minutes at 37° C, after which the cells were washed with cell culture medium.

# Quantification of cell membrane cholesterol content by filipin staining

Cells were grown to 80% confluence in MATTEK P35 glass-bottom dishes. Exogenous cholesterol:MβCD 120 μg/mL or an equal volume of medium was added as described above and incubated for 15 minutes at 37° C. Cells were washed with medium and fixed with 4% paraformaldehyde (PFA) in PBS for 15 minutes at 37° C. Cells were washed twice with PBS. PFA was quenched by addition of glycine to a concentration of 1.5 mg/ml in PBS for 15 minutes at room temperature. Cells were again washed twice with PBS. Cells were stained with 0.05 mg/ml filipin in PBS and incubated for 2 hrs at room temperature. Cells were washed twice with PBS and observed with a Zeiss LSM 880 microscope. Cell surface fluorescence was quantified using ImageJ software.

# Cell infection assay

GAS strain 854 was grown to mid-exponential phase, collected by centrifugation, suspended in DMEM, and inoculated at a multiplicity of infection of 0.2 onto the surface of confluent HAP1-Cas9 knockout or control cells in 24-well plates. Infected cells were incubated at 37° C in 5% CO2 for

2 hrs. Cells were washed twice with PBS and then incubated in DMEM with 1% FBS and 20 μg/ml penicillin overnight at 37° C in 5% CO2. Cell viability was determined 20-22 hrs post-infection by trypan blue exclusion (12).

# Protein labelling with Alexa Fluor 647

Expression and purification of SLO G395V G396V were described previously (4, 13). Purified protein was labelled with Alexa Fluor 647 according to the manufacturer’s recommendations (Alexa Fluor 647 NHS ester, Invitrogen). Labelled protein was protected from light, flash-frozen in liquid nitrogen, and stored in aliquots at -80°C.

# Reconstitution of membrane glycosphingolipids

A431*ugcg* cells were seeded in opaque-walled 96-well plates at a density of 10,000 cells per well. Cells were allowed to adhere for 20 hrs in DMEM at 37° C in 5% CO2. Cells were washed 3 times with prewarmed DMEM without serum. Individual glycosphingolipids were suspended at a range of concentrations in DMEM containing 5 μM defatted-bovine serum albumin (dfBSA, Sigma). Glycosphingolipids were equilibrated for 5 mins at 37° C and then added to the cells and incubated at 37° C in 5% CO2 for 30 mins. Cells were washed twice with 0.2 μM dfBSA in DMEM and then tested for susceptibility to SLO-mediated cytotoxicity by incubation with 0.6 nM SLO as described above.

# Incorporation of GM1 analogs and SLO binding

A431 UGCG KO cells were grown on Mattek glass bottom dishes. Cells were washed 3 times with DMEM without phenol red (Gibco). GM1 C18:0 or GM1 C18:1^Δ9^ complexed to dfBSA in a 1:0.75 ratio were added to the cells at 5 or 0.5 µM, respectively, in DMEM without phenol red. Equal incorporation of GM1 C18:0 and GM1 C18:1^Δ9^ was validated by FACS using fluorescently

labeled cholera toxin B-subunit: cells were lifted using trypsin, chilled to 4° C for 10 min, and then incubated with 10 nM cholera toxin B-subunit fluorescently labeled with Alexa Fluor 488 (Thermo Scientific) for 15 min at 4° C. Cells were collected by centrifugation at 500 g for 5 min. Supernatant was discarded and cells were washed in 1 ml PBS before FACS analysis. Data were analyzed using FlowJo software. For SLO binding, cells were incubated for 20 min at 37° C. Cells were washed 3 times with PBS and then chilled to 4° C for 15 min. SLO G395V G396V-Alexa Fluor 647 was added at 20 to 200 nM for 15 min at 4° C. Cells were fixed after SLO addition using 0.2% glutaraldehyde and 4% formaldehyde in cytoskeletal buffer (10 mM MES, 150 mM NaCl, 5 mM EDTA, 5 mM MgCl2, and 5 mM glucose) for 15 min at room temperature. Cells were washed with 3 times with PBS and then imaged by STORM microscopy (14).

# Super-resolution microscopy

Super-resolution microscopy was performed using an ELYRA7 and 8 microscope. For STORM, fixed cells were submerged in 50 mM TRIS, pH 8, 10 mM NaCl, GLOX (0.5 mg/ml glucose oxidase, 40 µg/ml catalase, 10% glucose with 100 mM mercaptoethanol (final concentrations)). Images were reconstructed using Zeiss ZenBlack software (pixel size 97 nm, photoelectrons per A/D count 0.46, base level (A/D counts=97, according to manufacturer), and localizations were exported to text files to be analyzed using ImageJ plugins ThunderSTORM and decodeSTORM (15, 16). For each image, one region of 2 by 2 µm was chosen for analysis. Ripley’s K function analysis was performed to assess SLO cluster radius (17).

# Statistical analysis

Statistical significance of differences between groups was evaluated using one-way ANOVA with Tukey’s or Dunnett’s multiple comparison test (SLO binding and inhibition assays, SLO clustering analysis), two-way ANOVA with Tukey’s or Dunnett’s multiple comparison test (SLO cytotoxicity assays), or one sample *t* and Wilcoxon test (GAS infection assays).

# References

1. Gryllos I, Grifantini R, Colaprico A, Cary ME, Hakansson A, Carey DW, Suarez-Chavez M, Kalish LA, Mitchell PD, White GL, Wessels MR. 2008. PerR confers phagocytic killing resistance and allows pharyngeal colonization by group A Streptococcus. PLoS Pathog 4:e1000145.
2. Love JF, Tran-Winkler HJ, Wessels MR. 2012. Vitamin D and the Human Antimicrobial Peptide LL-37 Enhance Group A Streptococcus Resistance to Killing by Human Cells. mBio 3:e00394-12.
3. Schmieder SS, Tatituri R, Anderson M, Kelly K, Lencer WI. 2022. Structural basis for acyl chain control over glycosphingolipid sorting and vesicular trafficking. Cell Rep 40:111063.
4. Velarde JJ, O'Seaghdha M, Baddal B, Bastiat-Sempe B, Wessels MR. 2017. Binding of NAD(+)-Glycohydrolase to Streptolysin O Stabilizes Both Toxins and Promotes Virulence of Group A Streptococcus. mBio 8:e01382-17.
5. Michos A, Gryllos I, Hakansson A, Srivastava A, Kokkotou E, Wessels MR. 2006. Enhancement of streptolysin O activity and intrinsic cytotoxic effects of the group A streptococcal toxin, NAD-glycohydrolase. J Biol Chem 281:8216-23.
6. Shalem O, Sanjana NE, Hartenian E, Shi X, Scott DA, Mikkelson T, Heckl D, Ebert BL, Root DE, Doench JG, Zhang F. 2014. Genome-scale CRISPR-Cas9 knockout screening in human cells. Science 343:84-87.
7. Joung J, Konermann S, Gootenberg JS, Abudayyeh OO, Platt RJ, Brigham MD, Sanjana NE, Zhang F. 2017. Genome-scale CRISPR-Cas9 knockout and transcriptional activation screening. Nat Protoc 12:828-863.
8. Martin M. 2011. Cutadapt removes adapter sequences from high-throughput sequencing reads. EMBnetjournal 17:3.
9. Langmead B, Salzberg SL. 2012. Fast gapped-read alignment with Bowtie 2. Nat Methods 9:357-9.
10. Wang H, Baldwin KA, O'Sullivan DJ, McKay LL. 2000. Identification of a gene cluster encoding Krebs cycle oxidative enzymes linked to the pyruvate carboxylase gene in Lactococcus lactis ssp. lactis C2. J Dairy Sci 83:1912-8.
11. Team RC. 2020. R: A language and environment for statistical computing., *on* R Foundation for Statistical Computing. https://[www.R-project.org/.](http://www.R-project.org/) Accessed 15 May.
12. Cywes Bentley C, Hakansson A, Christianson J, Wessels MR. 2005. Extracellular group A Streptococcus induces keratinocyte apoptosis by dysregulating calcium signalling. Cell Microbiol 7:945-55.
13. Velarde JJ, Piai A, Lichtenstein IJ, Lynskey NN, Chou JJ, Wessels MR. 2022. Structure of the Streptococcus pyogenes NAD(+) Glycohydrolase Translocation Domain and Its Essential Role in Toxin Binding to Oropharyngeal Keratinocytes. J Bacteriol 204:e0036621.
14. Arumugam S, Schmieder S, Pezeshkian W, Becken U, Wunder C, Chinnapen D, Ipsen JH, Kenworthy AK, Lencer W, Mayor S, Johannes L. 2021. Ceramide structure dictates glycosphingolipid nanodomain assembly and function. Nat Commun 12:3675.
15. Thompson RE, Larson DR, Webb WW. 2002. Precise nanometer localization analysis for individual fluorescent probes. Biophys J 82:2775-83.
16. Song Q, Wu C, Huang J, Zhou Z, Huang Z-L, Wang Z. 2023. DecodeSTORM: A user- friendly ImageJ plug-in for quantitative data analysis in single-molecule localization microscopy. Journal of Innovative Optical Health Sciences 16:13.
17. Kiskowski MA, Hancock JF, Kenworthy AK. 2009. On the use of Ripley's K-function and its derivatives to analyze domain size. Biophys J 97:1095-103.
